# Prophylactic cranial irradiation for patients with small-cell lung cancer: a systematic review of the literature with meta-analysis

**DOI:** 10.1186/1471-2407-14-793

**Published:** 2014-10-31

**Authors:** Wenwen Zhang, Wenjing Jiang, Linlin Luan, Lili Wang, Xiangrong Zheng, Gongchao Wang

**Affiliations:** School of Nursing, Shandong University, Jinan, 250012 China; School of Medicine, Shandong University, Jinan, 250012 China

**Keywords:** Prophylactic cranial irradiation, Small-cell lung cancer, Meta-analysis

## Abstract

**Background:**

Small cell lung cancer (SCLC) accounts for about 13% of all lung cancer cases. Small cell lung cancer (SCLC) accounts for about 13% of all lung cancer cases. The purpose of the present article is to assess the role of prophylactic cranial irradiation (PCI) in small cell lung cancer (SCLC) by performing a systematic review of the randomized trials published in the literature.

**Methods:**

Randomized controlled trials were identified that compared brain metastases incidence and overall survival between PCI and No PCI in patients with SCLC. Search strategies were limited to the English language and to articles published since 1997, and included: databases searched from 1997 to March 2013 –CINAHL, Embase, Medline, Web of Science, and CENTRAL. Methodological quality was assessed with the Jadad scale. The main end points were brain metastasis and survival.

**Results:**

The review identified 5 trials, although few were of high quality. Two trials reported the one-year incidence of brain metastasis. PCI reduced the incidence of brain metastasis in one year, with a pooled relative risk of 0.45 (95% CI, 0.35 to 0.58; P < 0.00001). Four trials described the one year survival rate. The combined result revealed a significant (P = 0.01) survival benefit in the group assigned to PCI as compared with the control group, with a pooled relative risk of 0.87 (95% CI, 0.79 to 0.97). Three trials reported the three-year survival rate. The combined result revealed a great significant (P < 0.00001) survival benefit in the PCI group as compared with the No PCI group, with a pooled relative risk of 0.87 (95% CI, 0.83 to 0.91). the Five-year survival rate was compared in four trials Compared with the No PCI group, the PCI group had a significant (P < 0.00001) survival benefit with a pooled relative risk of 0.92 (95% CI, 0.88 to 0.95).

**Conclusions:**

The present systematic review indicates that PCI decreases brain metastases incidence and that PCI improves survival in SCLC patients. Prophylactic cranial irradiation should be part of standard care for all patients with small-cell lung cancer who have a response to initial chemotherapy, and it should be part of the standard treatment in future studies involving these patients.

**Electronic supplementary material:**

The online version of this article (doi:10.1186/1471-2407-14-793) contains supplementary material, which is available to authorized users.

## Background

Small cell lung cancer (SCLC) accounts for about 13% of all lung cancer cases [1]. SCLC is characterized by rapid doubling time, early dissemination and high sensitivity to chemotherapy and radiotherapy [2–4]. Chemotherapy has improved short-term survival, but long-term survival remains disappointing. The 2-year survival rate among patients with extensive small-cell lung cancer was 1.5% in 1973 and 4.6% in 2000 [1]. SCLC has a propensity to metastasize to the brain. About 10% of the patients initially present with brain metastases. The two-year cumulative risk rises to ≥50% [5] and brain metastases are found in up to 65% of patients at autopsy [6]. The median survival time after brain metastases diagnosis is 4 to 5 months. Because the blood–brain barrier has been considered to protect the central nervous system (CNS) from most cytotoxic agents and as SCLC is very radiosensitive, the role of PCI has been studied in several trials [7].

PCI was first tested for patients with SCLC in the 1970s following the recognition that the blood–brain barrier appeared to restrict the penetration of most chemotherapeutics into the brain leaving it as a sanctuary site for relapse [8]. The first trial about PCI demonstrated substantial reductions in brain metastases [9]. The results of the randomized trials show that PCI reduces the frequency of brain metastases although survival is not consistently improved. Some data suggest that the gain in survival is restricted to patients in complete remission (CR). A published meta-analysis [9] of PCI for SCLC in patients with CR after chemotherapy has analyzed the data of 7 randomized studies (including one abstract and one unpublished study) concerning a total of 987 patients (526 treated with PCI and 461 controls). The relative risk (RR) of death in the treatment group as compared to the control group was 0.84 (95% confidence interval CI: 0.73 to 0.97; *p* = 0.01). PCI decreased also the cumulative incidence of brain metastases (RR: 0.46; CI 95%: 0.38-0.57; *p* < 0.001).The results of these trials consistently revealed a significant decrease in the incidence of brain metastasis [10, 11]. The purpose of the present article is to assess the role of PCI in SCLC by performing a systematic review of the randomized trials published in the literature.

## Methods

### Trials selection

Studies eligible for inclusion were randomized controlled clinical trials fully published in journals and those identified from other sources (abstracts and proceedings of relevant meetings) for which full details are available from investigators from 1997 to March 2013. Patients of any age had randomly assigned to receive PCI or not.

We searched CINAHL (from 1981), Embase (from 1980), Medline (from 1966), Web of Science (from 1966), and CENTRAL (from 1977) to present, using search strategies developed with the support of an information specialist that included exploded MeSH terms. Please see the Additional file 1.

Two independent reviewers read titles, abstracts, and full text papers and applied the inclusion criteria. Two reviewers independently extracted data from included full text papers. In case of incomplete or unclear data on study design and clinical outcome, authors were contacted. Discrepancies were resolved by a third referee.

### Methodological assessment

Methodological quality of randomised controlled trials was assessed in accordance with a well-established, validated scale developed by Jadad and colleagues [12]. A Jadad score was calculated using 4 elements of consideration. 1) Was the randomization scheme described and appropriate? 2) Was the method of double-blinding appropriate? 3) Was the method of Concealment of allocation appropriate? 4) Was there a description of dropouts and withdrawals? The possible range of scores was from 0 (weakest) to 7 (strongest). Any study with a Jadad score below 3 was considered to be of poor quality.

Four authors independently evaluated the quality of the trials. Using a standardized protocol and reporting form, they extracted data on the subjects’ characteristics at baseline and data on the clinical outcomes. Any disagreement was resolved through group discussion.

### Statistical analysis

Statistical analysis was performed using the Review Manager 5.2. Relative risk (RR) and 95% confidence intervals (95% CI) were used as summary statistics. The pooled relative risk was calculated by using a fixed-effect model with the Mante-Haenszel method and the Breslow-Day test was used to examine the statistical evidence of heterogeneity across the studies (p < 0.1). The Der Simonian and Laird random effect model was additionally applied to calculate pooled relative risk in case of significant heterogeneity across studies.

Sensitivity analyses were performed to assess the effects of selected measures of study quality. The influence of each study was estimated by deleting each in turn from the analysis and noting the degree to which the effect size and significance of the treatment effect changed. This analysis was performed for each study outcome. We considered a study influential if the exclusion of it changed our conclusion or the effect estimate by at least 20%.

## Results

A total of 5 randomised trials published between 1997 and 2012 were found to be eligible for this review. This study includes two of the studies included on the Auperin meta-analysis (Gregor et al., Laplanche et al.) [9]. In addition, this meta-analysis also includes 3 newer studies that have been published well after the Auperin meta-analysis. Their main characteristics are summarized in Table 1. The total number of eligible patients included was 1601; the number of patients by study ranged from 51 to 739 patients. The time of median follow-up ranged from 9 to 72 months.

**Table 1:** **Characteristics of the six trials included in the meta-analysis**

Author	Year	Age (Years)		Median follow-up (month)	Total dose/No. of fraction (Dose/Fraction)	No. of patients
		PCI	No PCI			
Gregor [11]	1997	60 (37–79)	61 (28–76)	18	36 Gy/18 (2 Gy)	314
					24 Gy/12 (2 Gy)	
Laplanche [13]	1998	58	57	-	24-30 Gy/8-10	211
Cao KJ [14]	2005	54.69 ± 7.56	55.63 ± 7.29	60	36-40 Gy/18-20	51
Slotman [15]	2007	63 (37–75)	63 (39–75)	9	20-30 Gy/5-12	286
Schild [8]	2012	63 (34–79)	63 (37–80)	72	30 Gy/15 (2 Gy)	739
					25 Gy/10 (2.5 Gy)	

### Methodological quality of included studies

Initial agreement among reviewers on the overall methodological quality was 90%, and after the consensus meeting, no disagreement persisted. The results of the methodological quality assessment are presented in Table 2. All the studies were considered to be of low quality by scoring 2 or 3.

**Table 2:** **Jadad quality scores of the six trials included in the meta-analysis**

	Randomization	Concealment of allocation	Double blinding	Withdrawals and dropouts	Total
Gregor 1997 [11]	1	1	0	0	2
Laplanche 1998 [13]	1	1	0	1	3
Cao KJ 2005 [14]	1	1	0	0	2
Slotman 2007 [15]	1	1	0	1	3
Schild 2012 [8]	1	1	0	0	2

### Brain metastasis

**Figure 1:**
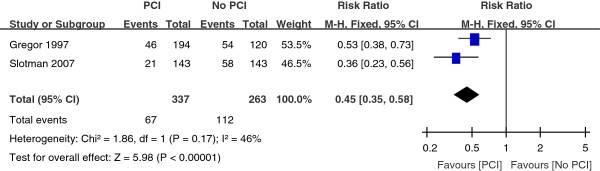
**Relative-risk plots for brain metastasis of 1 year.**

Three trials reported the one-year incidence of brain metastasis. But we included two eligible trials. If Cao KJ’s study was included, the heterogeneity would exist among the trials. So we excluded it. As shown in the Figure 1, PCI reduced the incidence of brain metastasis in one year, with a pooled relative risk of 0.45 (95% CI, 0.35 to 0.58; P < 0.00001).

### Overall survival

**Figure 2:**
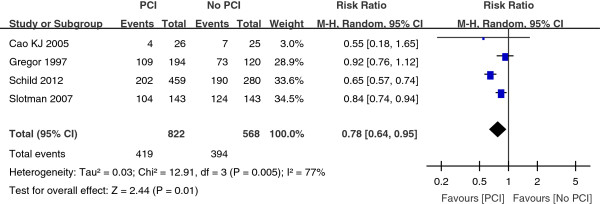
**Relative-risk plots for death of 1 year in patients with SCLC.**

**Figure 3:**
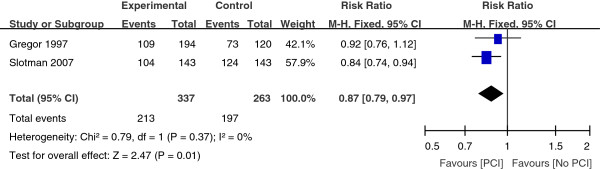
**Adjusted relative-risk plots for death of 1 year.**

One year survival rate were described in four trials between the PCI group and the No PCI group. We combined the result by using a fixed-effect model with the Mante-Haenszel method. The combined result showed that the heterogeneity existed among the trials (Figure 2). So we excluded two trials [8, 14]. The combined result revealed a significant (P = 0.01) survival benefit in the group assigned to PCI as compared with the control group, with a pooled relative risk of 0.87 (95% CI, 0.79 to 0.97) (Figure 3).

**Figure 4:**
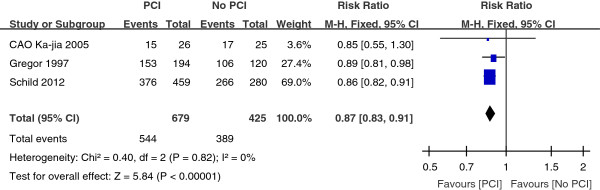
**Relative-risk plots for death of 3 year.**

**Figure 5:**
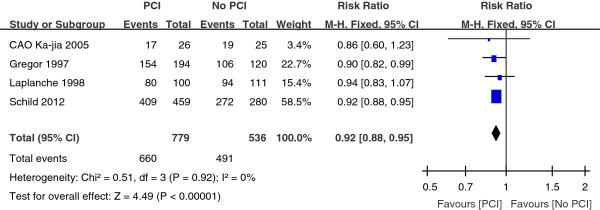
**Relative-risk plots for death of 5 year.**

Three trials with a total of 1104 patients reported the three-year survival rate. As depicted in Figure 4, the combined result revealed a great significant (P < 0.00001) survival benefit in the PCI group as compared with the No PCI group, with a pooled relative risk of 0.87 (95% CI, 0.83 to 0.91). As shown in Figure 5, the five-year survival rate was compared in four trials with a total of 1151 patients. Compared with the No PCI group, the PCI group had a significant (P < 0.00001) survival benefit with a pooled relative risk of 0.92 (95% CI, 0.88 to 0.95).

## Discussion

Our meta-analysis, by pooling five randomized studies that assessed PCI in 1941 patients with small-cell lung cancer, revealed a positive effect of PCI. As shown by the meta-analysis, irradiation not only significantly reduced the risk of brain metastasis, as previously revealed in individual trials, but also improved overall survival. These results confirm that PCI prevents and does not simply delay the emergence of brain metastases.

Brain metastasis is one of the most important causes of treatment failure in patients with SCLC. Most brain metastases occur within 2 years of diagnosis [16–22]. And median time to relapse in the brain is about 5.7 to 11.7 months [17–21]. Among the patients who survived for more than 2 years, about 50 percent of patients had brain metastasis [23]. With longer survival, brain metastases are being observed more often. PCI is effective in reducing the incidence of brain metastasis of SCLC. Several randomized studies showed that PCI reduced the rate of brain metastases in the patients with SCLC who achieved CR [10, 24, 25].Our meta-analysis also revealed that PCI reduced the incidence of brain metastasis within one year, with a pooled relative risk of 0.45 (95% CI, 0.35 to 0.58; P < 0.00001). Combining with previous studies, we concluded that PCI reduced the incidence of brain metastases. Thus, patients with SCLC should be treated with PCI to reduce the incidence of brain metastasis.

In addition to thoracic radiotherapy, PCI has been shown to improve survival in SCLC patients. More recently, Patel et al. provided supporting data that PCI was associated with better survival of LSCLC patients [26]. This large retrospective analysis included 7995 patients with limited staged-SCLC. The 5-year survival was 11% without PCI versus 19% with PCI (P < 0.001). PCI also improves survival rate for the majority of extensive staged-SCLC patients. Slotman et al. conducted a randomized trial of PCI in extensive staged-SCLC patients who had had any degree of response to chemotherapy [15]. Patients were randomly assigned to undergo PCI or the control group. The cumulative risk of brain metastases within 1 year was 14.6% in the PCI group and 40.4% in the control group (HR, 0.27; P < 0.001). PCI was associated with an increase in median survival from 5.4 to 6.7 months after randomization. The 1-year survival rate was doubled at 27.1% in the PCI group and 13.3% in the control group (P = 0.003). Auperin et al. published a meta-analysis which included data from seven randomized prospective studies which compared PCI with no PCI after a CR was achieved [9]. The 3-year survival rate was 5.4% better for those who received PCI at 20.7% compared with 15.3% for those who did not receive PCI (P = 0.01). While a 5.4% improvement in survival appears small, this reflects a 35% increase in 3-year survival and is clinically meaningful. According to our meta-analysis, the combined result for one-year survival rate revealed a significant (P = 0.02) survival benefit in the group assigned to PCI as compared with the control group, with a pooled relative risk of 0.87 (95% CI, 0.79 to 0.97) (Figure 3). For the three-year survival rate, the pooled relative risk was 0.87 (95% CI, 0.83 to 0.91). And the pooled relative risk was 0.92 (95% CI, 0.88 to 0.95) for five-year survival rate. The findings from the present analysis provide further support to the result that survival was significantly better for patients with SCLC who received PCI compared with No PCI.

There are some toxic events after the long-PCI. The most common acute toxic events were fatigue (30% of patients in the standard-dose group versus 34% in the higher dose group), nausea or vomiting (23% versus 28%), and headache (24% versus 28%) [27]. Neurologic abnormalities seemed to be very common in long-term survivors with SCLC and may be more prominent in patients having received high-doses chemotherapy or treated with large brain radiotherapy fractions. In the 1980s, several nonrandomized studies found neuropsychological impairment and abnormalities on CT scans of the brain that were potentially related to PCI [28–32], and a recent study of patients treated by PCI and concomitant chemotherapy suggested that this combination had a negative effect on cognitive function, which was assessed at the end of treatment [33]. In this systematic review, we did not discuss the toxicities of long-term PCI. The problem of neuropsychological toxicity remains unclear, leading to controversy about the indications of PCI in SCLC. So further studies about neuropsychological toxicity of the long-term PCI need studied by the researchers in the future.

## Conclusions

The present systematic review indicates that PCI decreases brain metastases incidence and that PCI improves survival in SCLC patients. Prophylactic cranial irradiation should be part of standard care for all patients with small-cell lung cancer who have a response to initial chemotherapy, and it should be part of the standard treatment in future studies involving these patients.

## Electronic supplementary material

Additional file 1: **PRISMA–Flow Diagram.** It’s a PRISMA flow diagram 278 for this study. (DOC 60 KB)
