# Supplementary material for: Prophylactic cranial irradiation for patients with small-cell lung cancer: a systematic review of the literature with meta-analysis
Source: BMC Cancer. 2014 Oct 31;14:793. doi: 10.1186/1471-2407-14-793 (PMC4232715; doi:10.1186/1471-2407-14-793)
Supplement: Supplementary file 1 — Additional file 1: PRISMA–Flow Diagram. It’s a PRISMA flow diagram 278 for this study. (DOC 60 KB) [file 12885_2013_4984_MOESM1_ESM.doc]

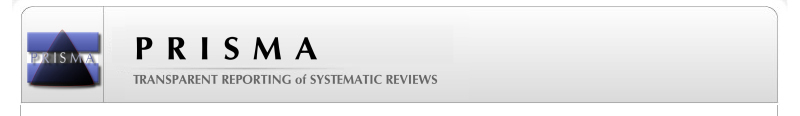
**PRISMA 2009 Flow Diagram**

**Screening**

**Included**

**Eligibility**

**Identification**

Records identified through database searching
(n = 226)

Additional records identified through other sources
(n = 2)

Records after duplicates removed
(n = 227)

Records screened
(n = 29)

Records excluded
(n = 198)

Full-text articles assessed for eligibility
(n = 14)

Full-text articles excluded, with reasons
(n = 15)

Studies included in qualitative synthesis
(n = 5)

Studies included in quantitative synthesis (meta-analysis)
(n = 5 )
